# The RAP study, report 3: Discoloration of the macular region in patients with macular neovascularization type 3

**DOI:** 10.1111/aos.14866

**Published:** 2021-04-05

**Authors:** Bilal Haj Najeeb, Gabor G. Deak, Ursula Schmidt‐Erfurth, Bianca S. Gerendas

**Affiliations:** ^1^ Vienna Reading Center Department of Ophthalmology Medical University of Vienna Vienna Austria

**Keywords:** age‐related macular degeneration, dense exudates, intraretinal haemorrhage, macular neovascularization, retinal angiomatous proliferation

## Abstract

**Background/Aims:**

To explore whether the existence and pattern of distribution of macular haemorrhage or exudate can be valuable diagnostic markers for macular neovascularization type 3 (MNV3) in patients with neovascular age‐related macular degeneration.

**Methods:**

Eighty‐three eyes of 83 consecutive treatment naïve patients with stage 3 MNV3 were enrolled. The diagnosis was based on fluorescein angiography (FA) and optical coherence tomography (OCT). Subretinal and intraretinal haemorrhage and dense exudates were evaluated on colour fundus photography. Fluorescein angiography (FA) images and OCT scans were used to identify the axial location of the haemorrhage. 83 patients with MNV1 and 83 with MNV2 were included as two control groups.

**Results:**

In the MNV3 group, 62 (75%) eyes had intraretinal haemorrhage and 52 (63%) had dense exudates. 73 (88%) eyes had intraretinal haemorrhage and/or dense exudates. 41 (49%) had both pathologies. The intraretinal haemorrhage was flame shaped over the lesion and punctate or semi‐punctate further away from it and directed to the fovea. No subretinal haemorrhage was noticed. In the MNV1 and MNV2 groups, 11 (13%) and 24 (29%) eyes had subretinal haemorrhage or dense exudates, respectively. No intraretinal haemorrhage was seen in the two control groups. The prevalence of exudates and haemorrhage (irrespective of its location) was greater in MNV3 than in MNV1 or 2 (p < 0.0001).

**Conclusion:**

The existence and pattern of distribution of intraretinal haemorrhage is pathognomonic of MNV3. It makes (alone or with dense exudates) the diagnose MNV3 possible using fundoscopy or colour fundus photo and without further diagnostic expenditure.

## Introduction

Early diagnosis of neovascular age‐related macular degeneration (nAMD) is important for clinical practice and ongoing retinal research, in particular in macular neovascularization type 3 (MNV3), previously known as retinal angiomatous proliferation (RAP), where massive exudative maculopathy occurs leading to a devastating and irreversible visual loss if treatment is delayed (Viola et al. 2009). When treated in time, it responds very well to anti‐vascular endothelial growth factor (VEGF) therapy (Hemeida et al. 2010). Since MNV3 is bilateral in almost all cases (Gross et al. 2005), close monitoring of the partner eye is also important once the diagnosis is set in the first eye (Wong et al. 2020). On the other hand, to date many well‐known multicentre clinical trials that have included patients with nAMD have tended to restrict the diagnosis to classic and occult choroidal neovascularization (CNV) dependent on the dynamic characteristics of the lesion on fluorescein angiography (FA) only (Heier et al. 2012; Busbee et al. 2013; Dugel et al. 2020). Consequently, clinicians or graders in reading centres when determining eligibility for trial inclusion may underestimate MNV3 or even incorrectly include up to about one fourth of MNV lesions as classic/occult CNV although no clear choroidal onset can be seen (Massacesi et al. 2007; Spaide 2018). In addition, further examination is usually required to diagnose MNV3, including indocyanine green angiography (ICGA) or optical coherence tomography angiography (OCTA) (Massacesi et al. 2007; Rouvas et al. 2010). The ‘hot spot’ which is a common feature of MNV3 on ICGA is also found in 30% of eyes with MNV1 (Ravera et al. 2016). Optical coherence tomography angiography (OCTA) is still not available in most medical centres and both ICGA and OCTA have not yet been implemented as standard examinations in clinical nAMD studies. Therefore, a simple noninvasive and reliable method of diagnosis is crucial to identify MNV3 at baseline.

In this study, we investigate the incidence and pattern of distribution of dense exudates, intra‐ and subretinal haemorrhage in eyes with MNV3 and compare the outcomes with those in eyes with MNV1 and 2 to draw conclusions on the origin of MNV3 from colour fundus photography (CFP) only.

## Materials and methods

All three patient groups were participants in prospective randomized multicentre double blinded studies to explore the efficacy of anti‐VEGF treatments in nAMD. They had newly diagnosed treatment naïve macular neovascularization secondary to nAMD without confounding diseases such as diabetic retinopathy or retinal vascular occlusion. Eighty‐three eyes of eighty‐three consecutive patients with treatment naïve solitary stage 3 MNV3 were enrolled in this analysis. The same number of eyes with MNV1 and MNV2 each was included as two control groups. Colour fundus photography with a minimum resolution of 1536 × 1536 pixels, FA images with a minimum resolution of 1024 × 1024 pixels and OCT raster scans taken according to predefined imaging protocols for each OCT device were obtained at baseline in all patients. Ethical approval for this *post hoc* analysis was obtained from the Ethics Committee of the Medical University of Vienna and for the core trials; it was obtained from the institution’s review board of each of the participating centres that took part in the multicentre trials. The images were graded at the Vienna Reading Center (VRC). We only evaluated the screening visit for this *post hoc* analysis.

**Fig. 1:**
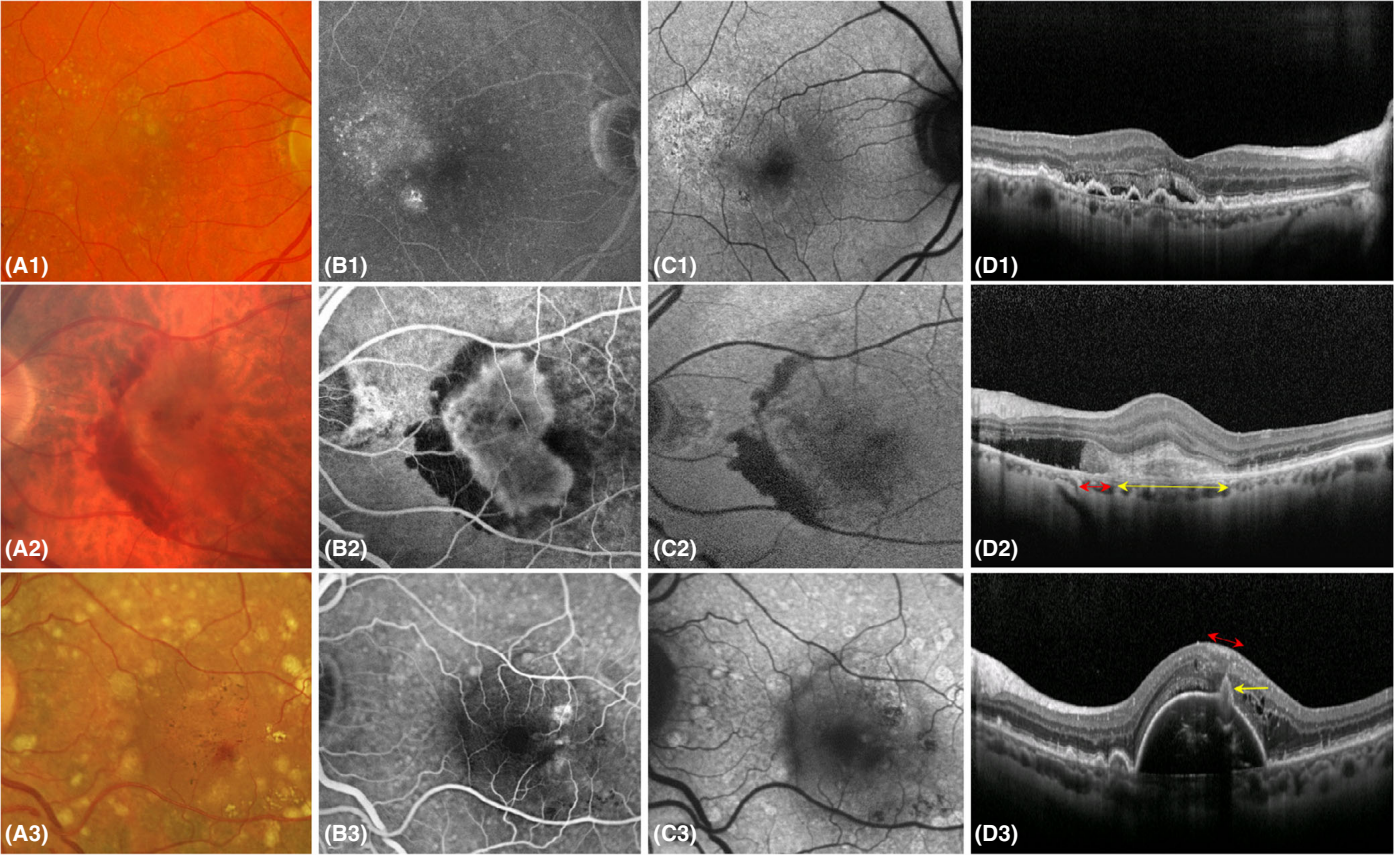
Multimodal imaging of all three types of macular neovascularization (MNV): Panel 1 illustrates an eye with MNV1, where drusen maculopathy is found on colour fundus photography (CFP) (1A); a speckled hyperfluorescence from an ill‐defined lesion is seen on late‐phase fluorescein angiography (FA), oval‐shaped changes in the retinal pigment epithelium on autofluorescence (AF) (1C) and multiple pigment epithelial detachments (PED) and subretinal hyporeflective fluid on an optical coherence tomography (OCT) B‐scan (1D). Panel 2 shows an eye with MNV2 as a grey discoloration in the central macula with a surrounding semicircular subretinal haemorrhage on CFP (2A). A well‐demarcated hyperfluorescence outlines the MNV2 lesion and complete hypofluorescence due to the blockade of the subretinal haemorrhage is seen on early‐phase FA (2B), a moderate and complete hypoautofluorescence due to the MNV2 lesion and haemorrhage respectively on AF (2C) and subretinal hyperreflective material (yellow double headed arrow), haemorrhage (red double headed arrow) and hyporeflective fluid on an OCT B‐scan (2D). Panel 3 shows an eye with MNV3 as superficial retinal haemorrhage obscuring part of the feeding arteriole with surrounding red pinpoint haemorrhages and yellow hard exudates on CFP (3A). In addition, there are brown pigment clumpings, and drusen maculopathy. Leakage arising from the lesion as hyperfluorescence close to the superficial haemorrhage and a central round hypofluorescence referring to a PED on early‐phase FA (3B). A round moderate hypoautofluorescence at the location of the PED and complete hypoautofluorescence at the location of the haemorrhage, exudates and pigments are seen on AF (3C). The superficial haemorrhage presents as thickened nerve fibre layer (red double headed arrow) causing shadows on the underlying neovascular mass (yellow arrow), which in turn disrupts the fibrovascular PED on an OCT B‐scan (3D).

We used the following grading criteria to diagnose the three different MNV types using FA and OCT only (without CFP):

MNV1 (occult): A fibrovascular pigment epithelial detachment (PED) on FA and OCT or late leakage as a speckled hyperfluorescence from an ill‐defined lesion on FA (Fig. 1).

MNV2 (classic): A lesion with bright well‐defined boundaries on an early‐phase image of FA which leaked profoundly later, and a hyperreflective confined mass overlying the RPE with the absence of a PED on OCT (Fig. 1).

**Fig. 2:**
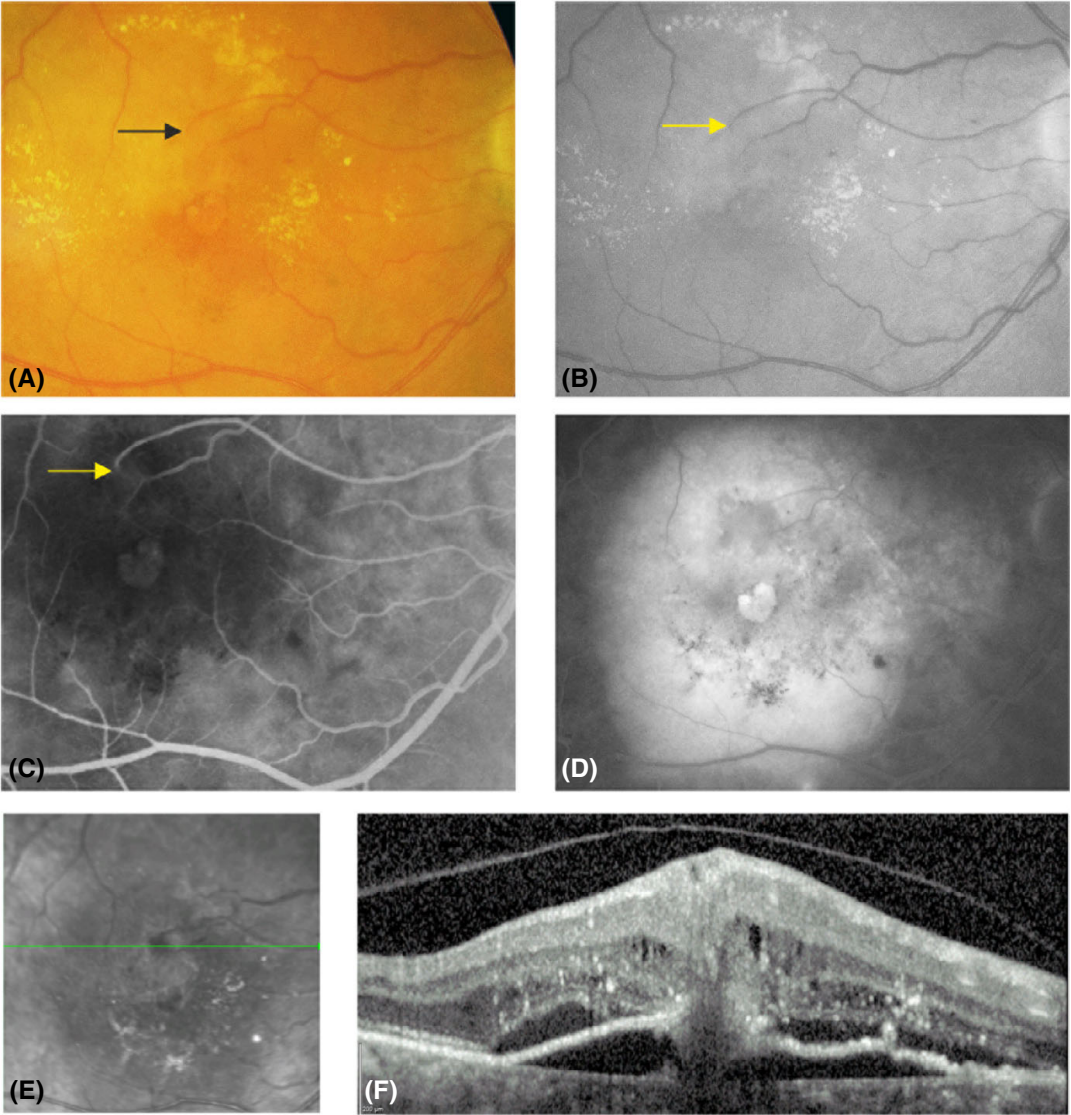
Macular neovascularization type 3 (MNV3) with dense exudates: on colour fundus photography circular dense exudative changes can be demonstrated and an arterial–venular anastomosis can be seen (black arrow) (A). The same findings can also be identified on the red‐free image (yellow arrow) (B). The arterial–venular anastomosis on an early‐phase image of fluorescein angiography (FA) (yellow arrow) presents a heart‐shaped foveal leakage (C). The late‐phase FA image shows extensive leakage and a fibrovascular pigment epithelial detachment (PED) (D). The corresponding optical coherence tomography B‐scan shows characteristic changes of MNV3: disruption of the PED with an overlying hyperreflective mass which extends deeply to anastomose with the choroidal neovascularization (E, F).

**Fig. 3:**
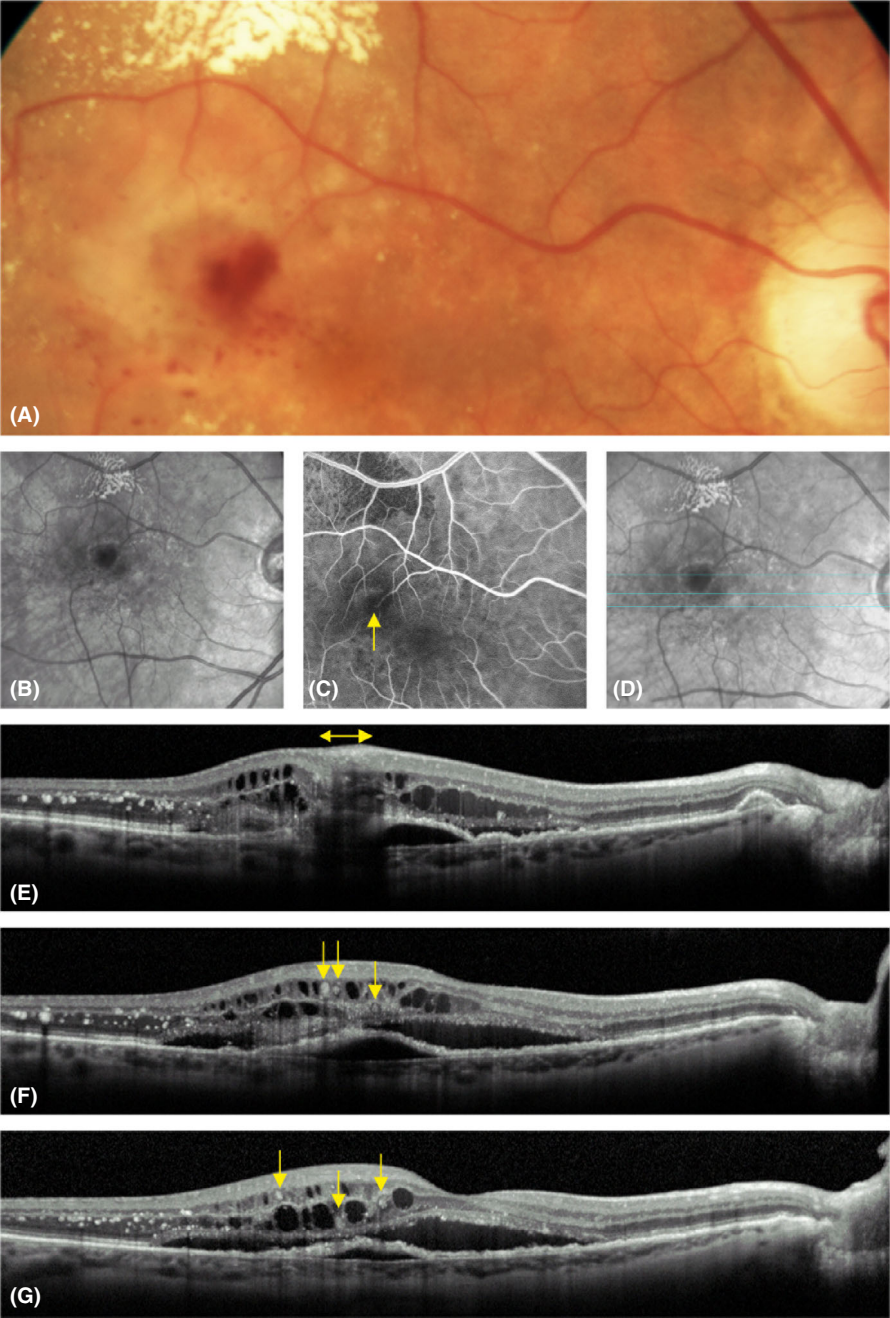
Macular neovascularization type 3 with dense exudates and intraretinal haemorrhage: the colour fundus photo shows exudates in yellow and intraretinal haemorrhage in red. Note that the small haemorrhages tend to occur close to the fovea more than any other area around the lesion (A). The same findings can be seen on the red‐free image, where exudates present in white and haemorrhage in black (B). An early‐phase image of fluorescein angiography (FA) presents the abrupt diving of a retinal arteriole (yellow arrow) surrounded by intraretinal haemorrhage (C). The corresponding near infrared image presents the location of three (upper, middle and lower) optical coherence tomography (OCT) scans (D). The upper OCT b‐scan through the big haemorrhage close to the lesion demonstrates its location in the inner retina (yellow double ended arrow) causing shadows beneath (E). The two (middle and lower) OCT B‐scans through small haemorrhages far from the lesion show their intracystoid location (yellow arrows) (F–G). Note the numerous exudates present as hyperreflective foci in the OCT B‐scan (E–G).

**Fig. 4:**
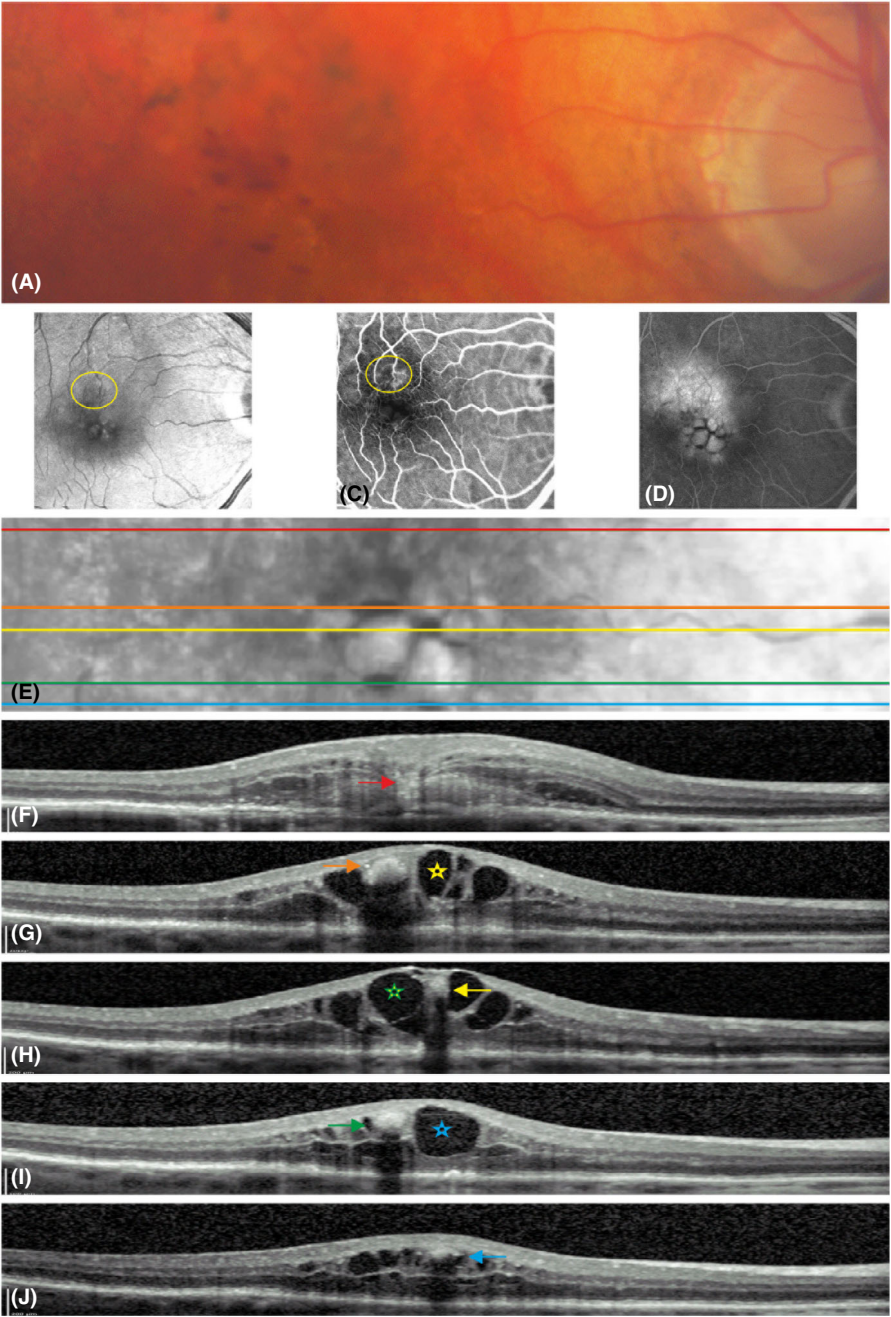
Macular neovascularization type 3 with intraretinal haemorrhage: the colour fundus photo demonstrates intraretinal haemorrhage as multiple red points close to the fovea (A). The red‐free image shows the arteriolar–venular anastomosis (yellow ring) (B). An early‐phase fluorescein angiography (FA) image presents focal hyperfluorescence in the area of the anastomosis (yellow ring) (C). The late‐phase FA image shows the central leakage and the intracystoid dot and boat‐shaped haemorrhage (D). Note the characteristic formation of level due to the incomplete accumulation of blood into the cavity of the cyst. A part of the corresponding near infrared image presents the location of five optical coherence tomography scans (OCT) scans cutting the haemorrhages (E). An OCT B‐scan (red line on E) shows the disrupted pigment epithelial detachment by a thick strand of a hyperreflective angiogenic network as a hallmark of MNV3 (red arrow) and a massive retinal swelling (F). Four sequent b‐scans (orange, yellow, green and blue lines on (E) further away from the lesion across intracystoid haemorrhages (arrows with the same colours) with shadow effect (G–J). Each sedimented hyperreflective haemorrhage (yellow green and blue arrows) (H–J) is located at the bottom of a corresponding hyporeflective cystoid formation (stars with the same colours) in the overlying OCT scans (G–I).

MNV3: Focal extrafoveal hyperfluorescence usually at an arteriolar–venular shunt or at a retinal arteriole diving abruptly deep into the outer retina on an early‐phase image of FA, and a hyperreflective complex upon an interrupted PED accompanied by intraretinal cystoid fluid on the corresponding OCT B‐scans (stage 3 according to Su et al.) (Ravera et al. 2016; Su et al. 2016) (Figs 1, 2, 3, 4).

**Fig. 5:**
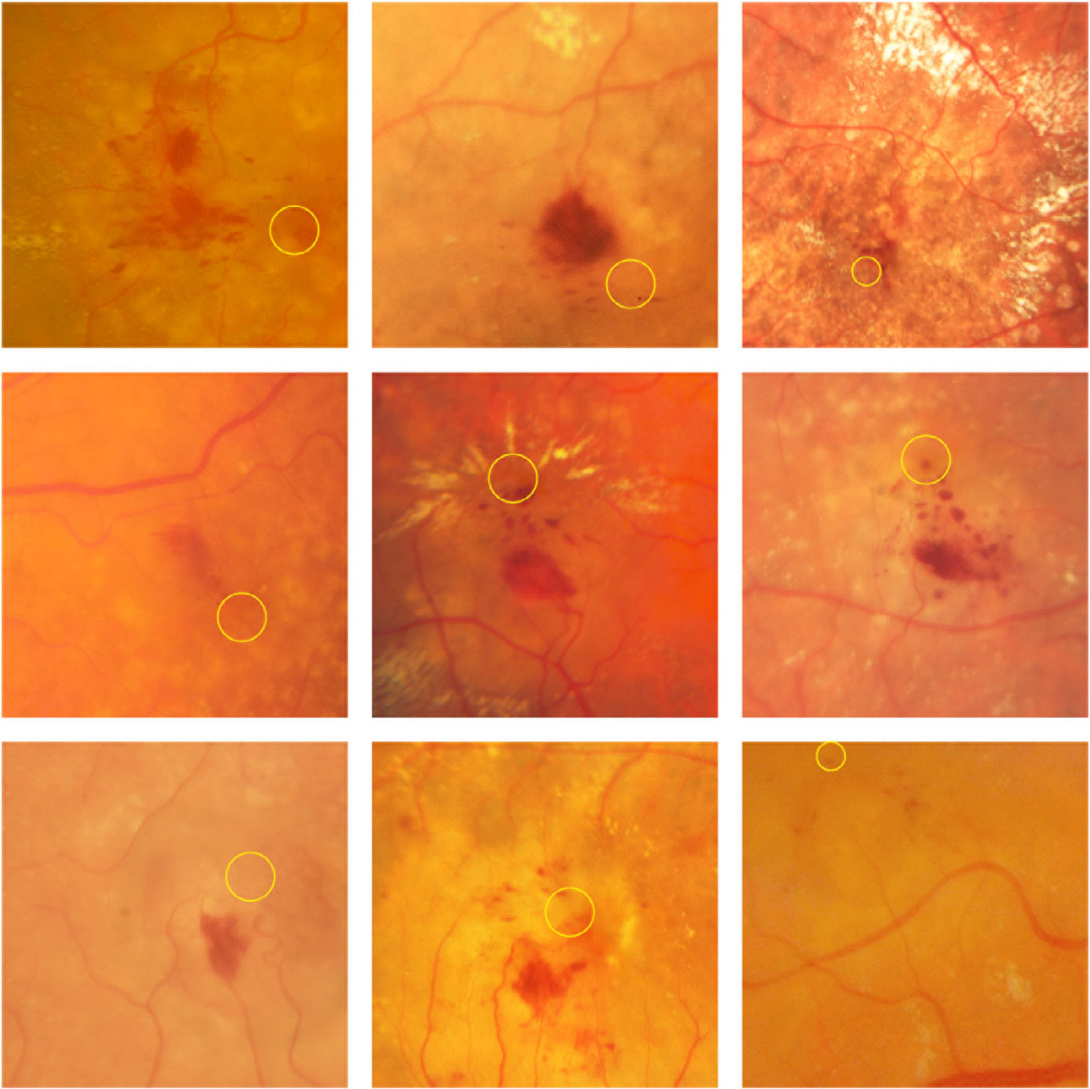
Colour fundus photos of intraretinal haemorrhage in patients with newly diagnosed macular neovascularization type 3. The thick big haemorrhage is usually solitary, flame‐shaped and located in the nerve fibre layer over the lesion, whereas the small punctate and semi‐punctate haemorrhages are numerous, located further away, reside in the cystic formations and directed to the fovea (yellow ring). In some cases, only one pattern of both haemorrhages was found. Note the presence of exudative changes surrounding the haemorrhages and the absence of subretinal haemorrhage which is typically seen in macular neovascularization types 1 and 2.

The type of lesion was determined at the VRC by two independent certified and experienced graders. Only in the case of a discrepancy between the readers, the decision was taken by their supervisor (retina specialist). Then, the existence of dense hard exudates as yellow confluent plaques around the lesion on CFP was explored (Figs 1, 2, 3, 5). The presence of the intraretinal haemorrhage was investigated as a dot‐ or splinter‐shaped red coloration which could block the retinal vessels on CFP depending on its axial extension in the retina, and dark areas on FA that could still be identified to the end of the angiogram (Figs 1, 3, 4, 5). The subretinal haemorrhage was assured as an amorphous homogenous red coloration usually located around the CNV under the retinal vasculature on CFP. On fluorescein angiography (FA), the subretinal haemorrhage was defined as a black area which was covered partially or completely by overlying leakage during the angiogram. In all cases, the corresponding OCT scans were used to verify the exact axial location of the haemorrhage (Figs 1, 3, 4).

## Results

### Patients with MNV1

Eleven eyes (13%) showed subretinal haemorrhage or dense exudates. Subretinal haemorrhage only was noticed in three eyes (4%). Eight eyes (10%) were associated with dense exudates. None of the eyes presented with both features. Haemorrhages did not present any special pattern on CFP. They were covered partially or completely by overlying hyperfluorescent leakage on the late‐phase images of FA. None of the eyes with MNV1 had an intraretinal haemorrhage (Fig. 1; Table 1).

**Table 1:** The distribution of subretinal haemorrhage (SRH), intraretinal haemorrhage (IRH) and dense exudate (DE) in eyes with the three types of macular neovascularization (MNV).

MNV Type	Eyes	SRH	IRH	DE	SRH & IRH & DE	SRH or IRH or DE
MNV1	83	3 (4%)	0 (0%)	8 (10%)	0 (0%)	11 (13%)
MNV2	83	19 (23%)	0 (0%)	6 (7%)	1 (1%)	24 (29%)
MNV3	83	0 (0%)	62 (75%)	52 (63%)	41 (49%)	73 (88%)

### Patients with MNV2

Twenty‐four eyes (29%) showed a subretinal haemorrhage or exudates. Only one eye presented (1%) with both pathological entities. Six eyes (7%) had dense exudates. A subretinal haemorrhage was seen in 19 eyes (23%), more often than in eyes with MNV1 (p < 0.0001). The subretinal haemorrhage took a partial or complete circular shape around the fibrovascular lesion (Fig. 1). As in the MNV1 group, none of the eyes had an intraretinal haemorrhage (Table 1).

### Patients with MNV3

None of eyes presented with a subretinal haemorrhage. Otherwise, 62 (75%) eyes had an intraretinal haemorrhage (Figs 1, 3, 4, 5). The prevalence of haemorrhage (regardless of its location) in MNV3 was greater than in MNV1 or 2 (p < 0.0001). Moreover, 52 (63%) eyes had dense exudates (Figs 1, 2, 3, 5). The incidence of dense exudates in MNV3 was also higher than in MNV1 or 2 (p < 0.0001). Seventy‐three (88%) eyes had an intraretinal haemorrhage and/or dense exudates on CFP. Forty‐one (49%) had both pathologies (Figs 1, 3, 5) (Table 1). The prevalence of red (haemorrhage regardless of its location) and yellow (exudates) colours was higher in MNV3 than in MNV1 or 2 (p < 0.0001). The intraretinal haemorrhage above or in close proximity to MNV3 was a profuse splinter shaped. However, haemorrhage located further away from the lesion was multiple punctate or semi‐punctate and directed to the fovea on CFP (Figs 1, 3, 4, 5). On the corresponding OCT scans the splinter shape form of intraretinal haemorrhage was harboured in the inner retinal layers, mainly in the nerve fibre layer. Whereas punctate and semi‐punctate haemorrhages resided in intraretinal cysts, respectively (Figs 3, 4). As intraretinal haemorrhage was present in the inner retina, it was easily recognizable as a well‐defined black area surrounded by hyperfluorescent leakage on late‐phase FA images (Fig. 4). Dense exudates were distributed around the lesion, but further away than haemorrhage (Figs 1, 2, 3, 5).

## Discussion

Our *post hoc* analysis shows that an intraretinal haemorrhage is pathognomonic for treatment naïve MNV3 lesions. The haemorrhage is solid and looks flame shaped in the centre of the MNV3, whereas further away a punctate or semi‐punctate pattern is typical. No subretinal haemorrhage was identified in the study group. Moreover, haemorrhages and exudates are predominantly found in MNV3 than in the other two MNV types.

In the following sections, we discuss each pathologic entity in turn followed by a conclusion on the possibilities of diagnosing MNV3 using such discoloration on CFP.

The first pathologic entity we found was intraretinal haemorrhage, which was very common and pathognomonic for MNV3 (Figs 1, 3, 4, 5). This could be due to the distinctive pathogenesis of this type, where the neovessels are initially intraretinal. Then, they extend deeply into the outer retina before continuing under the retina and eventually beyond the retinal pigment epithelium to anastomose with the underlying CNV (Yannuzzi et al. 2001). The vertically oriented fragile neovessels and their limited ability to stretch to cope with the increasing retinal oedema may make them more susceptible to bleed in the inner retina causing a thick splinter‐shaped haemorrhage close to the lesion.

In recent reports, a schematic drawing of MNV3 was presented where the distant punctate and semi‐punctate haemorrhages, contrary to our findings, were located outside cystic formations and no adjacent retinal oedema was illustrated (Spaide 2019; Spaide et al. 2020). A local effect of VEGF was proposed as a cause for the development of these haemorrhages (Spaide 2019). Whereas, our outcome using multimodal imaging demonstrated that these haemorrhages occur in oedematous retinal tissue and typically filled pre‐existing cystoid formations (Figs 3, 4). Furthermore, the intraretinal location of haemorrhage was distinguishable as marked small black spots surrounded by white leakage (oedema) on late‐phase FA images (Fig. 3). Also, our observation that distant haemorrhages tend to occur close to the fovea where the cystoid formations are usually prominent refers that the cystoid formations have a major role on its occurrence (Figs 3, 4) (Liakopoulos et al. 2008; Brar et al. 2010; Ravera et al. 2016). Therefore, we suggest that distant haemorrhages are likely to originate from the extensive stretch forces the cysts apply on the capillaries running in the inner retina. Thus, these haemorrhages are expulsive in nature rather than a slow chronic leakage. In addition, we found that oedema was extended at least to the area of distant haemorrhage, as they were located exclusively within cystoid formations (Figs 3, 4). Similar topographic approaches have been used in several studies to explain the causes and characteristics of different retinal diseases (McBain et al. 2011; Haj Najeeb et al. 2012,; Mauschitz et al. 2012; Haj Najeeb et al. 2017; Haj Najeeb et al. 2020).

The second pathologic entity we investigated was dense exudates, which were also more common in MNV3 than in the other two MNV types. This is also an aid to outweigh the presence of MNV3 when examining maculae of patients with nAMD. The increased lipids in blood serum could be a reason because patients with this type of lesion have hypertension and are usually older than those with MNV1 or 2 (Caramoy et al. 2014). The increased permeability of retinal anastomosis compared with choroidal neovessels could be another factor. The anatomical location of the anastomosis above the retinal pigment epithelium (RPE) could be another reason why the exudates are easy to identify compared with MNV 1, where the RPE could obscure the underlying exudates during fundus examination.

The easy recognition of one or both aforementioned signs (intraretinal haemorrhage and exudates) would be helpful to diagnose this type of MNV using fundoscopy in clinical practice or CFP at reading centres. Further expensive and invasive examinations such OCTA or ICG could be avoided. Moreover, using only such entities to differentiate the types of MNV opens the gate for promising diagnostic methods that would be easier and faster. In time, retina specialists could differentiate MNV types using artificial intelligence‐assisted cameras as currently in diabetic retinopathy (Abràmoff et al. 2018).

Subretinal haemorrhage secondary to nAMD is usually associated with a poor prognosis compared to intraretinal haemorrhage (Bennett et al. 1990). Therefore, our outcome is also important for graders in central reading centres who sometimes need to determine whether haemorrhage is intra or subretinal using CFP only.

The subsequent visual deterioration could be caused through the barrier effect of blood, the later traction in the outer retina or the toxicity of blood to photoreceptors (Hochman et al. 1997). Interestingly, we found that none of the patients with MNV3 had subretinal haemorrhage. A previous work showed that subretinal haemorrhage may develop in up to 20% of patients with MNV3 receiving anti‐VEGF treatment (Lee et al. 2017). This discrepancy between baseline and follow‐up might be explained by the choroidal component, which is likely responsible for this type of haemorrhage, being less responsive to anti‐VEGF therapy than the retinal one. We also found that the incidence of subretinal haemorrhage was statistically significantly higher in MNV2 than in MNV1. This correlates with the fact that MNV2 presents a more advanced stage of CNV due to the penetration of the RPE and subretinal space. The shape of the haemorrhage as a partial or complete ring around the CNV is also helpful to diagnose MNV2 on CFP (Fig. 1).

In many studies, diagnosis of MNV3 has been attempted using mono‐ or multimodal imaging (Gass et al. 2003; Kim et al. 2014; Ravera et al. 2016). In one study the incidence of intraretinal haemorrhage with soft drusen was estimated as 33% only (Kim et al. 2014). In contrast, our study reveals either intraretinal haemorrhage or dense exudates, or both, have a much higher diagnostic value (Table 1). Moreover, the aforesaid study did not include either a control group or explore the prevalence of dense exudates, which were of great value for the diagnosis of MNV3 in two thirds of the cases in our study. In addition, the authors classified intraretinal haemorrhage into intra‐ and preretinal haemorrhage, as they did not use OCT to check the correct location of the haemorrhage. A misdiagnosis could have arisen from the shape of the intraretinal haemorrhage, which can look like preretinal haemorrhage. When a haemorrhage fills the cystoid formation incompletely, it shows level or boat‐shaped haemorrhage similar to that seen in typical preretinal haemorrhage (Fig. 4).

One of the limitations of our study is that in accordance with our methods, we included only patients with stage 3 of MNV3. Otherwise, intraretinal haemorrhage has also been reported in stages 1 and 2, in 43% and 39% of cases, respectively (Kim et al. 2014). A further shortcoming of our study is the absence of demographic information of our patients because all patients’ details were pseudonymized according to the study protocols of the prospective multicentre studies graded at the VRC. As a reading centre, we are always masked to demographic data of patients which clearly is a limitation and should be investigated in further analyses of patients with different types of MNV. Also, patients with subfoveal or widespread subretinal haemorrhage had been excluded according to the eligibility criteria of the clinical studies from which we derived our data. Therefore, polypoidal choroidal vasculopathy, which frequently presents with a diffuse subretinal haemorrhage including the fovea, was not equally explored in our study.

In conclusion, in addition to the unique characteristics of MNV3 previously explored in our first two reports (Haj Najeeb et al. 2020; Haj Najeeb et al. 2021), we investigated more morphological features that help retina specialists, researchers and graders at reading centres to identify this lesion using fundoscopy or CFP and without the need of further diagnostic expenditure.
